# Using “ways of thinking and practising” to analyse final year medical student reflections and underlying concepts in preparedness for practice

**DOI:** 10.3389/fmed.2025.1577327

**Published:** 2025-06-02

**Authors:** Rachel Leyland, Hilary Neve, Elizabeth Drake, Tracey Collett

**Affiliations:** Peninsula Medical School, Faculty of Medicine and Dentistry, University of Plymouth, Plymouth, England, United Kingdom

**Keywords:** ways of thinking and practising, preparedness for practice, medical education, healthcare education, medical students, reflection

## Abstract

**Introduction:**

Ensuring that medical students are prepared for future practice is challenging for all medical schools. Most studies of preparedness involve newly qualified doctors and use quantitative methods such as self-report surveys focused on defined competencies, and often find graduates are unprepared for the complex and challenging areas of real-world practice. Qualitative methods, using conceptual ideas linked to learning such as ‘Ways of Thinking and Practising’ (WTP) are little explored in this area but could offer rich and useful insights about graduates’ preparedness. WTP recognises that, in addition to specific knowledge and skills, students need to understand the complex links between theory and practice and what it means to be part of their disciplinary community, in terms of culture, values and ways of seeing and being in the world. This study explored the written reflections of final year medical students on the threshold of practice, as they looked back at reflections from their previous years of study. It aimed to identify disciplinary WTP described by students and gain insights into their developing grasp of these.

**Methods:**

Thirty six reflections were analysed and a thematic analysis undertaken, using WTP as a sensitising concept.

**Results:**

Six inter-related WTP were identified. Illustrative quotes are provided which demonstrate how grasping each WTP involved students making connections between different elements of their learning and the ‘what’, ‘how’ and ‘why’ of knowing. Students reflected on the factors that facilitated their learning. Two resulting ‘changes in self’ were identified: a sense of confidence and self-efficacy, and a feeling of readiness for responsibility.

**Discussion:**

Grasping the WTPs identified may be a helpful part of preparing for practice, and understanding what facilitates this may be of use in informing future curricular design.

## Introduction

Ensuring that graduates are prepared for the complexity and unpredictability of future practice is a challenge for all medical schools, and how to achieve this remains uncertain. Even the term ‘preparedness’ has different meanings and the question ‘prepared for what?’, different answers. Stakeholders may conceptualise preparedness as short-term ‘hitting the ground running’ (1) or long-term, which includes practical and emotional aspects. Most studies employ quantitative methods (2) such as surveys of newly qualified doctors’ self-reported sense of preparedness with respect to defined competencies, or specific knowledge, skills and behaviours detailed in national competency frameworks such as the UK General Medical Council’s Outcomes for Graduates (3). Fewer studies have used qualitative approaches, such as audio-diaries and narrative interviews, yet these have the potential to provide rich, contextualised insights into graduates’ preparedness for practice (1, 4).

‘Ways of thinking and practising’ (WTP) (5) is a process-focused idea that recognises that, whatever their field, students need more than specific skills and knowledge to work effectively in the real world. They need to come to terms with those concepts which are central to the mastery of their discipline, as well as its culture, values, customs, forms of discourse, ways of acting and being, and how its members see and think about the world (6, 7). Moreover, they need to appreciate the complex links between disciplinary knowledge and practice, between ‘knowing what and knowing how,’ as well as ‘knowing why.’ (8). WTP have been little studied within medical education but may provide a useful conceptual framework (9) to understand those learning experiences that help prepare medical students for the complex, challenging and wicked problems they will encounter in healthcare (10–12). Identifying those WTP which enable students to ‘transition from thinking and practising like a medical student to thinking and practising like a [doctor],’ (13) (p. 182) and to understand what it means to be a doctor who works effectively as part of a healthcare community (6), could help medical schools design curricula and teaching which better prepares students for practice.

Most preparedness studies explore the experiences of newly qualified doctors; there is less literature about the views and perceptions of final year medical students as they approach the transition to clinical work. One such qualitative study, however, describes students bracing themselves for an abrupt ‘reality shock’ at transition (14) (p. 699). Coakley et al. found that, while some students were excited, many were anxious about assuming responsibility and apprehensive about the ordeals they expected to face, often planning dysfunctional approaches for dealing with those challenges (14). This aligns with Dornan’s description of newly qualified doctors experiencing a ‘baptism of fire’ (15). New graduates from our medical school regularly score highly in UK GMC preparedness for practice surveys and our study seeks to explore whether our final year students’ reflections provided any insights into this. Indeed, medical graduates enter an increasingly demanding and complex world of changing societal healthcare needs. Additionally, financial and staffing challenges in healthcare, and risks of burnout in junior doctors are increasing (1). Therefore, identifying factors which help prepare students for these issues is imperative.

### Study context

As part of their professionalism module, our medical school requires students to undertake reflective writing throughout their 5-year undergraduate programme. They reflect on a series of professionalism topics, linking these to their own individual experiences, most commonly those in the clinical setting. Toward the end of their final year, as part of a summative reflective writing assignment, students are asked to review, reflect on and refer to their personal written reflections from previous years and drawing on these, they respond to specific questions as below:

‘Review your previous reflections and drawing on these where appropriate, please reflect on the following (800–1,000 words):

- How have you changed since beginning at medical school?
- What is the most important concept you have understood since beginning medical school that enables you to think like a doctor?
- What is the most difficult concept about being a doctor you have encountered?’

These questions were adapted from a 2018 study exploring threshold concepts in third year paediatric clerks’ reflections (16).

### Study aims

This qualitative study aimed to:

- use WTP as a sensitising concept to analyse how final year students, through written reflection, articulate their evolving understanding of the challenges and underlying concepts which they consider important in their transition from student to doctor.

In doing so we also hoped to:

- identify WTP in medicine as described by fifth year medical students on the threshold of clinical practice.
- Gain insights into students’ developing grasp of these WTP during their medical course.
- Gain insights into students’ sense of preparedness for practice.

### Methodology

Starting in 2019 and following ethical approval, all year 5 students (*n* = 83) were invited to take part in this study in an address to the whole cohort. Students had already submitted and received results of their summative writing assessments and were asked to volunteer consent for their anonymised scripts to be analysed. Some research team members were known to them, but none had a direct role in assessing these students. All submissions were blinded and students were informed about confidentiality and that participation or non-participation had no influence on student progression. The School has an existing, confidential process to act on safeguarding or well-being concerns in the rare circumstances of these arising within students’ written reflections and researchers were aware of these. The research team comprised three clinical educators (two General Practitioners and an anaesthetist), one educationalist (a sociologist) and a research assistant with a background in psychology. The team independently read and re-read different selections of consenting students’ reflections and an initial coding scheme was developed (17). Coding frameworks were discussed and continuously adapted until consensus was reached. Data was coded using Lumivero (2020) NVivo 13 and an initial analysis undertaken to reveal themes, which were further discussed, negotiated and adapted by discussion (18). An initial study aimed to identify important and difficult concepts and personal change. During this analysis the research team decided that ‘Ways of Thinking and Practising’ would be an appropriate ‘sensitising concept’ and undertook this secondary analysis, reanalysing the data through the same iterative process to establish new themes (18). Making such a decision during data analysis is an approach known as theory informing inductive data analysis (9). Unlike definitive concepts, sensitising concepts are not prescriptive in nature (19) but can offer a sense of guidance, enhance sensitivity to nuances in the data and show how people give meaning to concepts in the context being studied (18). As the analysis progressed, the team continued to iteratively discuss, negotiate and refine the new themes emerging, whilst continuously reviewing them against the existing WTP literature and ensuring the themes remained grounded in the data.

## Results

**Figure 1:**
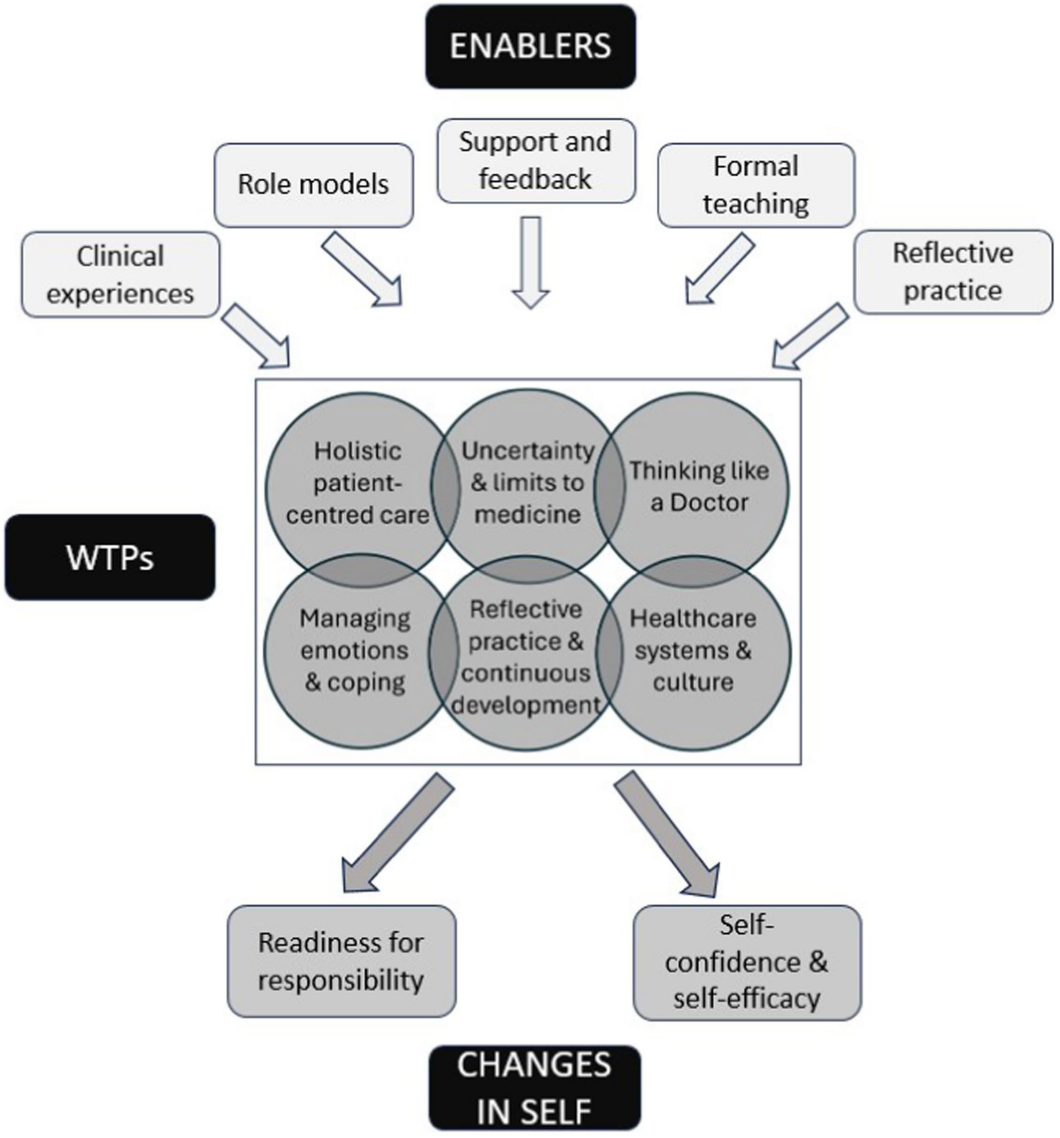
Ways of thinking and practising, with enablers and resultant changes in self.

36/83 students consented to their anonymised reflections being analysed. In all 36 reflections, students described coming to terms with new concepts during their undergraduate course, regularly linking their grasp of these concepts to their real-world experiences. As the analysis progressed it became apparent that many of the emerging themes fitted well to the descriptions of WTP within the literature. We identified six distinct WTP themes with some inevitable overlap between them (Figure 1). Students also often reflected on the factors that had helped them grasp these WTP, referring to the crucial role of clinical experiences, role models, the taught curriculum, support, and reflective practice. Alongside these six themes, we identified two ‘changes in self’ themes which appeared to be the result of grasping these WTPs, rather than WTPs in their own right (Figure 1). We considered WTPs to be discipline-specific approaches to understanding, reasoning and ways of being and seeing the world. They include frameworks and cognitive strategies (6). Separate to these ‘ways of being’, the ‘changes in self’ themes related to students’ descriptions of personal growth and changes in self-perception and confidence. These were more akin to the broader personal transformation that occurs through learning (20). These changes were often related to grasping the WTPs identified. While students largely appeared to see their new understandings and changes in themselves as positive and helpful, some described ongoing struggles, particularly in relation to the ‘systems and culture’ theme.

### WTP one: the importance of holistic, patient-centred care

In this major theme in the data, students described major changes over time in how they thought about their patients, with one student suggesting that the importance of putting *“the patient at the centre of their care”* was *“the biggest concept I have learnt since the beginning of medical school”* (S22). Sometimes this shift in thinking was influenced by clinicians. One student, for example, described regularly hearing the phrase *“treat the patient not the [scan/test result/bloods/imaging].”* (S28).

Aligned to this, grasping the importance of holistic care was a big shift for some students, who came to medical school expecting “*to learn all about diseases, the art of diagnosis and treatments from textbooks. It seemed a simple mathematical equation almost.*” This same student describes quickly learning “*that patients and their diseases are not separate entities. To be a good doctor, you must treat the patient as a whole. This starts with understanding the whole story.”*’ (S24).

Students described how learning about the biopsychosocial model, taking “countless histories,” having conversations with patients in different clinical settings, small group discussions and reflective practice had helped them appreciate complexity and question their pre-conceptions of patients. One student describes becoming aware of their own biases: *“I found it difficult initially when I saw patients walk through the door smelling of cigarettes or alcohol, or those grossly obese or using drugs. These are people, many with issues I cannot begin to imagine or as I have come to realise just daily struggles of real people.”* (S13). Another reflected on how *“behind every frustrating or rude patient, there is a reason. Maybe they are scared? Maybe they are tired of suffering from chronic pain? Maybe they have a mental health condition contributing to their behaviour?”* (S15).

Students commonly described how observing senior doctors communicate with patients, especially where time was limited, had helped them appreciate the importance of active listening, ensuring understanding and making decisions with patients. As one student reflected, “*It is easy for us to assume that the patient has understood and processed everything about an intervention within the 5 min they are seen within a ward round*.” (S16).

Another student considered how it was not always easy to respect patient autonomy, describing clinical experiences where “*what I think is best for the patient’s health contradicts what the patient themselves want. For example, an elderly lady diagnosed with cancer, who decided to go on holiday with her family before starting treatment. This delayed her treatment by a few weeks but brought her enormous happines*s.” (S32).

### WTP two: thinking like a doctor

In this significant theme, students described significant changes in their ways of thinking, including their development of clinical reasoning and the importance of drawing on underpinning scientific concepts and principles: “*I try to go back to the structure and function.…This helps me to understand how patients can develop particular symptoms but also helps me understand what investigations need to be done and why.”* (S10).

Students also reflected on their future role as decision-makers and the need to incorporate ethical principles, science and evidence into these decisions. One student described that *“From what I have seen in medicine, making complex decisions is the cornerstone of the profession…. Most importantly, it means I have had to learn to understand my own decision-making mechanisms, and to see what I use to justify my choices.”* (S36).

Some students described focusing on medical knowledge in their early years but that they now recognised the value of thinking as well as knowing. One student reflected on this, and the importance of remaining open-minded: “*Doctors need to be thinkers. I can so easily become pigeon-holed into a certain mindset or diagnosis without thinking about the wider picture or what else could be going on with a patient… …it puts me at risk of missing important signs and therefore mis-managing patients.”* (S4). Students highlighted how deep noticing, and thinking critically and ‘outside the box’ were often skills they had learnt from role models: “*The question ‘what else could it be?’ is one I have heard asked repeatedly by clinicians.”* (S31)

### WTP three: accepting and managing uncertainty and the limits to medicine

In this prominent theme, students reflected on how their views about uncertainty and ambiguity had evolved during their course. They commonly described struggling with these concepts at first, for example how patients may not “*present as they do in textbooks*.” (S10). One student reflected on how “*there can be multiple right answers, and with it the idea that people can come to different conclusions from the same information but both still be right in their circumstance”* (S36) and that this was the hardest concept they had encountered in medicine.

Others described becoming more confident with uncertainty, acknowledging, as one student stated, that it “*is a fundamental part of medicine.”* (S10). They linked these reflections to their approaching sense of responsibility, often suggesting approaches for managing uncertainty in the clinical setting. One student, for example, suggested “*It is about making sure that patients are safe—whether this is by safety netting, or escalating appropriately.”* (S7), while another described how “*Sometimes this involves being truthful with patients when you do not have the answers about a disease progress, treatment outcomes or prognosis.”* (S33).

Related to uncertainty, some students explored the nature of risk in relation to their future roles as a junior doctor “*such as discharge planning or deciding whether a patient should have a scan requested.”* (S19). They recognised the implications of the choices they would face, for example how, as one student reflected, “*the difference between sending someone home and admitting them for tests may be the difference between life and death.”* (S27).

Some students described how time on placements had changed their understandings around the limits to medicine and how they had come to recognise that they might not always be able to fix patients, predict or control all things. “*Before medical school, I used to think of doctors almost like gods. They could save everyone. Now, at the end of medical school, I know…how sometimes, you cannot do anything…Sometimes, you will be helpless. There are limits to medicine.”* (S34). They often linked this understanding to the ethical decision-making discussed in WTP2, with one student reflecting on how “*our attempts to fix…can do more harm than good and that the best thing may be to do nothing at all.”* (S14). Others’ reflections linked to WTP1 (patient-centred, holistic care), with one student highlighting the importance of “*being able to see the patient as a whole and understand the concept of a good death and producing the best outcome for them and their family holistically.”* (S23).

### WTP four: reflective practice and continuous development

Most students (*N* = 30) referred specifically to at least one past written reflection on experience, while the remaining six referred more generally to past reflections. Looking back at these appeared to help students see how they had changed, and how grasping a new concept could at times be sudden, associated with a specific experience or, more commonly, a more gradual shift in thinking, occurring over time and multiple experiences.

Looking back at their past reflections led students to reflect on past struggles, subsequent growth and transformed understandings and there was a strong sense of students seeking meaning.

Students often discussed how they had come to understand the value of reflection in medical practice and how their ability to reflect deeply and usefully had developed over their time at medical school. One student described comparing their recent reflective template to the first they completed in Year 3 and how their reflective skills had *“vastly improved.”* Previously, they described “*I would simply recount the details of an experience or superficially reflect on how it made me feel. Now I am able to reflect in much more detail, addressing how I felt, why I felt this way, the impact of the event and these emotions and also the knock-on effect on others such as colleagues and patients*.” (S12). A few students, however, still struggled with aspects of reflective practice, describing it, for example, as “*difficult and time consuming.”* (S21).

Several students highlighted how their new understandings of reflective practice informed their understandings of continuous professional development. As one student wrote: *“I used to see [reflection] as a tick box exercise with little meaning. Now I see it as a learning tool, a prompt and a platform to make change. I find reflection useful whether it be formal or informal. Simply sitting down quietly at the end of the day thinking about what went well and what could be improved is a useful and simple way of keeping on top of things and personal development.”* (S5).

Others recognised the importance of reflection and learning for healthcare with one, for example describing how, “*over the past few years I have become an independent learner… …I no longer learn for the sake of passing assessments, but I am now driven by the responsibility towards patients.”* (S24).

### WTP five: working within healthcare systems and culture

Of all the themes, this was the one where a number of students struggled, in two main areas. The first involved coming to terms with the demands of life as a junior doctor in the NHS. One, for example, reflected how they perceived “*a kind of hidden curriculum throughout medical school is that you will have to sacrifice things that you do not necessarily want to sacrifice in order to succeed.”* (S09). Another described warnings from doctors “*about the long antisocial hours and the fatiguing physical and emotional nature of the job*.” This same student, however, along with several others, felt this was balanced by the rewards of being a doctor, reflecting on how “*I have also seen how rewarding and stimulating medicine can be*.” (S01).

Just as in WTP3 where students came to acknowledge the limits of medicine, in this theme they also realised the limits of healthcare systems, and how bottlenecks and resource constraints can impact on the availability and quality of care they could provide. One student described *“…the stark pressures placed on the NHS due to patient demand and financial pressures is something that I see play out on a near daily basis.”* As a result, this student reflects, “*there’s a tension between trying to do tasks quickly and doing them in a safe manner that provides good quality patient care and also balance between caring for the patient in front of you at that particular moment against all the other patients under your team’s care.”* (S11). Coming to terms with this could be hard: “*I find this difficult to swallow. I want to give the best to patients without restriction and have had to realise that this is not always possible.”* (S18).

Students also highlighted a second area within this theme: the culture of healthcare including hierarchy, bullying and discrimination. Some linked this to learning about the hidden curriculum during their course. One student describes a series of experiences:

*“After being told in Orthopaedics that I have ‘tiny lady hands’ I have to remind myself that I will still be able to perform an examination of a hip joint. After seeing a consultant call a registrar a ‘baby doctor’ and demanding to speak to a ‘big boy’, I must remember that my clinical concerns as an F1 [newly qualified doctor] will be just as justified as if I were a consultant… …this is something I have reflected on in small groups and [written reflections]… …as soon as I understood there was a term for ‘hidden curriculum’ I became very passionate about respect in the workplace.”* (S25).

Again, some students struggled with this culture: “*I have come to dislike the attitude many doctors have towards one another, to get to [wherever] they want to go. We are indoctrinated to believe that to become the best you can be, you will need to put others down along the way.”* (S08). Others, however, sought to understand why these negative experiences might occur: “*I no longer find [‘scary seniors’] intimidating or take it personally anymore… …I recognise that people may act in certain ways irrespective of my own behaviour; perhaps they are sleep deprived, stressed, hungry or just like that sometimes*.” (S26).

A strong positive element of this WTP was how a culture of good teamworking, and their own role as part of a team, was seen to be key in providing good patient care and peer support: “*I have come to value the importance of reflection and debriefing with other team members, particularly after dealing with acutely unwell patients. Relationships with work colleagues are of paramount importance to both patient safety and clinician well-being.”* (S35). Others reflected on how teams may not always work effectively, with this student considering ways of addressing this: “*I learned about how easily poor teamwork can affect the atmosphere and proficiency of a team and that damaging a team’s cohesiveness can have knock on effects on patient care. Therefore for the sake of both my colleagues, patient experience and patient outcomes I will always try my best to be professional, calm and kind to my team members.”* (S12).

### WTP six: managing emotions and coping

Within this WTP students frequently discussed how they had previously struggled with upsetting situations and being able to ‘let go.’ They often made links to the WTP identified above, such as the emotions involved in maintaining patient centred, holistic care (WTP1) and accepting limitations in a challenging health system (WTP5). One student, for example, reflected how “*My view of death has evolved over the past years. In my year 4 [reflective template], I spoke about the first time I did CPR on a real patient… …who eventually died in front of us. I was in complete shock, felt disappointed in myself and very emotional when I came home.”* The same student then describes witnessing a recent patient death: *“This time round, there was less immediate emotion and a greater understanding of our limitations as a medical team.”* (S08).

Students often highlighted the importance of peers and team members in supporting them and of making time for reflection. One student, for example, looking back on their past reflections on the deaths of a newborn baby and a young woman, states: “*In both cases, only after talking to colleagues, did I feel unburdened.”* She also reflects *“where such discussion is not an option, I have found writing to help. When formally typing my [reflective template] I was able to analyse the situation from different perspectives which I found to be helpful. This is something that I can continue to carry out with future challenging patients.”* (S15).

Central to this WTP, students recognised that looking after a patient requires you to also look after yourself. They described a range of approaches for coping with the stresses of being a doctor, including eating well and making time to relax and exercise. Others described how time management techniques, such as allocating time to each task, would help when managing the demands of being a junior doctor: “*I have found passion in medicine and I am happy to give it time beyond my working hours, but I did not realise that if I was not organised or prepared then the amount of time needed would quickly increase*.” (S06).

Another student learnt when challenged by a small group peer for dismissing self-care: “*Though I was defensive at first, it became apparent that simple measures such as taking a five-minute break or ensuring I eat lunch would reap better long-term outcomes. Indeed, I have noticed that by integrating these steps into my day I am able to complete tasks more promptly as I am less fatigued.”* The student then reflects how “*This awareness has reiterated the importance of caring for oneself when caring for others*.” (S17).

A particular worry raised by a number of students was that, as they progressed through medical training, they might become desensitised and lose their compassion. One student reflects back on “*how much it terrified me coming home after spending the day in [the] oncology clinic breaking diagnoses of terminal cancers to patients without being as phased about it as I would normally be.”* (S02). This same student then discusses how using positive coping mechanisms, learning about intellectual empathy and having support systems in place have helped. The student reflected that: “*The most important concept that I have understood since beginning medical school is developing resilience without the loss of empathy,”* while acknowledging that “*It is imperative to be aware that resilience and empathy will both be indefinitely a work in progress, and I will continuously develop and work on protecting and nurturing both.”* (S02).

#### Changes in self

In addition to the WTP themes emerging from the data, we identified two consequential ‘changes in self’ themes (Figure 1).

### Change in self one: increased confidence and self-efficacy

Students frequently reported how they had often written about their anxiety, self-doubt or a lack of confidence in their early years’ reflections. Most, like this student, described how this had been replaced by an emerging sense of confidence and self-assurance. “*In reading my portfolio pieces from year one of medical school, it’s highly sobering to see just how much my confidence and outlook has developed… Not only am I now a team member who can offer her ideas and decisions to her team; I also have the confidence to advocate and work for the good of my patients as a foundation doctor.”* (S18).

Students often linked their growing confidence to coming to terms with the WTP above, such as accepting their limitations. For example, this student reflects: “*Nobody expects you to have all the answers, but if you can approach things calmly and sensibly you can hopefully get a good idea of what’s going on and make a suitable plan for the next step. I thought I had to be perfect and know everything and it [led] me to thrash around feeling helpless at times”* (S3) while another describes how “o*ver the past five years… I’ve learnt my limitations and when to seek help. Too much confidence can be dangerous.”* (S5).

Students frequently highlighted how clinical experiences including “*increased interactions with patients and healthcare professionals from a wide* var*iety of backgrounds”* had contributed to their “g*rowing sense of professional identity and responsibility”* (S16). They also cited academic support, including assessments during placements and feedback, alongside an increasing openness to learn from this, (WTP4). As one student reflects: “*When I receive feedback now, rat*her *than dwelling on the negatives and taking it personally, I now seek it out to find ways in which I could improve.”* (S24).

A few students described feeling less confident or sure of themselves in clinical situations. Most had considered how they would deal with this. One student, for example, reflecting on her anxiety when managing unwell patients reported “*[taking] steps to prepare myself for situations that I know I will find difficult.”* (S28). Another recognised that their lack of confidence stemmed from limited clinical experience and felt that “*as I build my experience as a junior my confidence in myself should continue to improve.”* (S21).

### Change in self two: readiness for responsibility

Closely linked to confidence was a sense of readiness for the responsibilities students would face as doctors and the implications of this. Most described this with cautious optimism, sometimes tempered by a degree of excitement or apprehension, and a sense of being “a*t the point where I feel ready to act as an F1 [newly-qualified] doctor.”* (S27). One student, for example, describes feeling *“as though I will be safe and have been given the skills required by the medical school to work safely and efficiently whilst also developing myself as a medical clinician and importantly a person next year too.”* (S13), while another reflects on the feeling of “*satisfaction that I get from doing the job well and can see the responsibility and purpose that I have craved for the last 4 years in the work.”* (S1) Students often reflected on the many connected elements of their learning that would enable them to manage this new responsibility. One, focusing on working in a multi-disciplinary team reflected how *“I have had to develop my appreciation of different perspectives, and challenged myself on my own perspectives. It has allowed me to face dilemmas and conflict and reflect on how I can manage it better in the future, knowing that there is not always a right answer.”* (S10). (links to WTP3 and WTP4).

A few students described a sense of trepidation about the degree of responsibility they would soon face in a less than perfect health system (WTP5): “*I have seen junior doctors breaking bad news to patients and they are often the only doctor on the ward for most of the day…and they are often responsible for dealing with the fallout from an underfunded and overwhelmed medical system.”* (S30). Others highlighted how mistakes and adverse outcomes would sometimes happen and that “*knowing that my actions will have a direct consequence on a patient’s outcome is generally quite frightening.*” (S29).

Another student, while accepting that “*mistakes are inevitable, and things will not always go the way you expect them to,”* reflected on how understanding this *“makes life that much easier as you will not be so hard on yourself as long as you remain transparent”* (S24) (link to WTP3). Other students pointed to the reassuring availability of support and advice if they feel out of their depth, while still accepting that in *“few months the bottom line stops with me.”* (S29).

## Discussion

This study of final year medical students’ reflections on learning and change during their undergraduate training, including the most important and most difficult concepts they had encountered or understood, provides valuable insights into their learning over time and readiness for practice. The use of WTP as a sensitising concept seemed to resonate with the data as well as facilitating analysis. It encouraged us to ask new questions of the data: to better understand how students described the concepts they identified as important or difficult, and how they attached meaning to these and to the changes they described in themselves. It helped us understand what appeared to help them to develop these new ways of thinking like, feeling and being a doctor (1).

The extensive preparedness for practice literature, involving a range of stakeholder groups, generally finds new graduates well-prepared for history taking, examination and simple diagnosis but often unprepared for unexpected and complex situations, knowing their limitations, prioritisation and the psycho-social elements of patient care (21). Medical board examinations in many countries worldwide typically focus on the acquisition of medical knowledge, or assess specific competencies in controlled settings (22). There is far less emphasis on learning and assessments which support students to develop the capabilities they need to provide holistic care to complex patients (23), to work effectively in a multi-disciplinary team (24) and to integrate skills and knowledge in different, authentic settings (22). Higher scores in the MCQ component of board exams, do not show correlation with reports of perceived preparedness (25). Rather, Chaou et al. argue, junior doctors struggle more with “real-life patient care” than medical knowledge (p. 7). This illustrates “the issue of preparedness is not clear-cut.” (26) (p. 9) WTP provides an opportunity to think in more expansive and contextualised ways about the curriculum, looking beyond disaggregated subject competencies and learning outcomes to consider how learners integrate these to gain bigger picture understandings and learn what it might mean to be part of their disciplinary community (6).

In this study, using WTP as a lens helped us to see how students made connections between different elements of their learning, including knowledge (knowing what), its application in practice (knowing how) and reflection and critical thinking (knowing why). For example, when reflecting on the WTP ‘holistic patient-centred care’ they critically reflected on why and how good communication skills, an awareness of personal bias, and the need to accept constraints such as time and workload, are all important in achieving this. When discussing decision-making (‘thinking like a doctor WTP’), students frequently considered its complexity and why, and how to use, critical thinking skills, underpinning scientific and ethical principles and consideration of different people’s perspectives. They often combined these with an acceptance of uncertainty, risk, not being able to fix everyone and knowing when to ask for help.

It is notable that the WTP and ‘changes in self’ themes identified in this study relate to, and may offer further insights into, the elements of Padley et al.’s work-readiness conceptual model (27). This was developed through a narrative view of the international literature on what constitutes work-readiness in medical graduates and includes ‘confidence,’ ‘capability,’ ‘responsibility,’ ‘reflexivity,’ and ‘resilience.’ The WTP identified in this study are also similar to threshold concepts previously reported in studies with medical students (16, 28). These include ‘Sometimes there is not a right answer,’ ‘Medicine is not black and white but grey and complex,’ ‘Treating the whole patient’ and ‘You cannot save everyone.’ In these studies, most students, who were in the earlier years of medical training, were still grappling with these concepts, recognising them as important but showing little evidence of transformation or irreversibility. Threshold concepts (TCs) are considered fundamental to the understanding of a discipline (29) and it is recognised that grasping them can take time as students traverse a ‘liminal space’ in learning (30), which usually involves struggle. While TCs describe transformative conceptual understandings, WTP are more concerned with how new and distinct disciplinary understandings can be applied in the workplace. However, there are parallels between the two (8). In our study, students commonly reflected on how grasping their new and complex ways of thinking and practising had involved a gradual shift over time. Evidence of change in how they saw themselves and their role as a doctor, as well as changes they described in their own behaviour, suggests our students had crossed conceptual thresholds and, in doing so, had grasped these new ways of thinking in practice. This may be fundamental to being prepared for practice.

Studies have found that graduates are often unprepared for the increase in responsibility and the psychological and emotional issues they will face (2, 4, 21). In this study some of our students recognised, but found hard, the idea that they would continue to encounter issues such as resource and time constraints, bullying and hierarchy, and that these could impact both on their own health and on patient care. Despite this most students described a feeling of confidence, readiness, and even excitement, for the imminent shift to much greater responsibility as a junior doctor. This is very different from the idea of final year students, braced for an abrupt ‘reality shock’ (14) (p. 699), where Coakley et al. describe students, balancing some positive anticipation with significant apprehension. Where students expressed a sense of readiness, it was often linked to a feeling of confidence, a term which can have different meanings. Confidence can mean ‘self-confidence,’ an optimism about their overall, global performance (31). This, in itself, can help a new graduate “[perceive themselves] as a doctor” and integrate better into the medical team, a “virtuous cycle” which improves the junior’s relationship with their supervisors (25) (p. 6). It may, however, carry with it the risk of students feeling more confident than their competence should allow (32). In this study, however, when students described feeling ‘confident,’ this aligned more closely with the notion of ‘self-efficacy’ i.e., a judgment as to their ability to address foreseeable specific situations (33). Self-efficacy has been associated with higher motivation and perseverance (34, 35) and may be an important element in students’ successful transition to clinical practice. Corfield et al. report up to a quarter of newly qualified doctors showed over-confidence in ethical decisions and suggests that preparedness involves recognition of difficulty and acknowledging doubt (32). Our data suggests that, in grasping the ‘acceptance and management of uncertainty and limitations’ WTP, students recognised both that they do not need to be perfect and that they would be continually learning. As one student explicitly stated, ‘too much confidence can be dangerous.’

In Ottrey et al.’s recent qualitative study, medical graduates described feeling unprepared in a number of areas including managing their time, responsibility, their own health, stress and emotions (4). Similarly, a 2017 GMC report cited significant challenges for newly qualified doctors, who felt stressed and unprepared for responsibility, multitasking, asking for help and unprepared for their own emotional responses (26). Therefore, it is relevant that in this study, one of the WTP identified was ‘managing emotions and coping.’ Within this theme students highlighted the importance of self-care, time management and the valuable support offered by their multi-disciplinary team. Wiese et al. highlight that managing emotions and self-care are ongoing skills, requiring further development in the new environment of clinical practice (36) (or as one of our students suggested “a work in progress.”) Ensuring that the facilitating factors identified in our study, including supportive teams, role models and helpful feedback, are in place is likely to be important in the newly qualified trainee environment.

Our study has allowed us to identify curricular activities and strategies which appear to aid development of key WTPs which may be related to students’ confidence in other areas with which newly qualified doctors struggle: managing complexity, ethical dilemmas, and maintaining holistic, patient centred care (28). Small group reflective discussion was frequently cited by students as helping them make sense of clinical learning experiences and make links to the theory learnt during teaching sessions. Indeed, reflection is a thread running through this study, both as a WTP itself and as a facilitator of learning and we have analysed this theme in more depth in a recent paper (37). Reflection has been identified as a critical element of health professions’ education (38). Such activities allow students to explore uncertainty and complexity. They also encourage the examination of the hidden curriculum, personal biases and thorny issues such as empathy, resilience, resource constraints, boundaries and limitations. This study may provide evidence of positive, potentially under-recognised, value of curricular activities focussed on reflection, which arguably aid development of key WTP.

In this study, role models were also cited as a strong facilitator of student learning and were largely, but not always, seen as positive. Several students in this study highlighted difficult issues they experienced in the clinical setting such as hierarchy, tribalism, poor team-working, and work-life conflicts, and made explicit links in their reflections to the ‘hidden curriculum’. All students in our school learn about, and reflect on examples of, the hidden curriculum during their course (39). Providing students with a tool to critically reflect on their clinical experiences and identify which modelled behaviours to emulate or not, may be particularly helpful in supporting the transition from student to doctor. Finally, our study also identified concepts such as heuristics, understanding decision making, and cognitive biases, as areas of significant learning. Curricular activities helping students understand this in theory and practice may help them in ‘thinking like a doctor’.

## Strengths and weaknesses

This study explores WTP, a conceptual framework little discussed in medical education, although it has been usefully explored in physiotherapy (40), history and biology (41). WTP can, it is suggested, be a useful approach for educators, particularly when looking beyond narrow competencies and learning outcomes to consider how best to prepare students for complexity of read real-world practice (42). While the WTP concept can be criticised for being open and hard to define (43), this is also true of the concept of preparedness’ (1). This may be why WTP seemed to resonate with our data, in that it provides a more sophisticated approach for understanding the nuanced and multifaceted nature of being work ready.

This study has a number of strengths; it builds on previous related studies and uses a qualitative methodology, under-represented in the preparedness for practice literature. The analysis of 36 students’ reflective writing offered rich and contextualised data. Quotes from all but one student have been used in this report, reflecting a breadth of data across the students studied and most students could have supplied several. The use of WTP offered us a new way to analyse the data and to study WTPs as they are learned (8). However, there are limitations. Some students’ reflections on how their understandings had developed and changed over time may have been subject to memory issues and hindsight bias, although the majority did draw on past reflections which had been written soon after their experiences. It is also important to note that students’ perceptions of readiness may change once practising and self-reported readiness can be shown to change in focus and degree during the first few months of work (4). The reflections of our final year students can be considered alongside findings that this cohort of students in this study did go on to score highly in the GMC trainee survey on perceptions of preparedness, reporting their perceptions some months after entering clinical practise (44).

All students were volunteers so we cannot be sure they are representative of the whole student cohort. A further limitation is that students’ reflective tasks were assessed by their tutor and this may have led some to ‘go through the motions’ (45) and tell their tutor what they thought they wanted to hear. However, students were assessed on the process, not the content, of their reflections, and would be aware from previous years that failing was rare and that they had the opportunity to re-write their reflection if needed. Another limitation is that students were not specifically asked about how prepared they felt for practice. The advantage is that students were not trying to write their reflections to fit this question, but it may be that important issues related to preparedness were not mentioned.

## Conclusion

This study suggests that Ways of Thinking and Practising may be a useful qualitative approach for medical educators to better understand how students view their learning and development during their undergraduate course. WTP may also offer educators and students a perspective on preparedness that, alongside competencies and skills, renders concrete the abstract activity of meaning-making. In this study, using WTP as a sensitising concept helped us to identify the conceptual understandings, personal changes and curricular elements that supported students towards thinking, acting like, and being a doctor. It enabled us to examine students’ capacity for metacognition, critical thinking, and above all their ability to make connections (for example between experience and knowledge and between different elements of the curriculum), and the relationship between this and their developing feelings of confidence and readiness for responsibility. In doing so this study may offer insights into how students make sense of medical practice at the point of entry into medicine.

While being competent may be an important element of preparedness, becoming aware of and comfortable with the disciplinary WTP identified in this study may also be key to work readiness. Quantitative analyses of preparedness disaggregate the experiences of new doctors into discrete measurable sets of competencies and skills. Results of such studies may contribute to educational practices that orient educators towards specific, focused behaviours at the expense of emphasising processes and ‘ways of doing.’ Unawareness of processual issues and their complexity in real time are likely to contribute to the initial shock reported by new doctors as they enter the workplace for the first time.

We cannot draw conclusions from this study as to how prepared our students really were, or whether they were able to implement the ideas they shared once working as junior doctors. The WTPs they report, however, may have aided their transition to their new role, where they later scored highly in self-reported preparedness, compared to peers from most other schools. Further studies, examining how WTP develop alongside their professional identity through undergraduate courses, and into the workplace beyond, could be valuable in establishing further insight into the enablers of preparedness. Conducting studies at other institutions could determine the generalisability of our findings for WTP in medicine and identify other curricular activities which appear to help students internalise these.

## Data Availability

The raw data supporting the conclusions of this article will be made available by the authors, without undue reservation.
